# Comparative Efficacy of Treatment Methods and Risk Factors for Treatment Failure in High-Grade Vaginal Intraepithelial Neoplasia: A Systematic Review and Meta-Analysis

**DOI:** 10.3390/cancers18081302

**Published:** 2026-04-20

**Authors:** Franciszek Ługowski, Magdalena Papież, Barbara Suchońska

**Affiliations:** 1 1st Department of Obstetrics and Gynecology, Medical University of Warsaw, 02-015 Warsaw, Poland; 2 Doctoral School, Medical University of Warsaw, 02-091 Warsaw, Poland

**Keywords:** vaginal intraepithelial neoplasia, vaginal cancer, human papillomavirus, cervical neoplasia, laser ablation, surgical excision

## Abstract

**Background:** High-grade vaginal intraepithelial neoplasia (VaIN 2+) is a rare condition with limited evidence to guide optimal management. This study aimed to evaluate the efficacy of various treatment strategies and identify clinical risk factors associated with treatment failure. **Methods:** A systematic literature search in PubMed, Scopus, Web of Science, and Cochrane databases was performed following PRISMA guidelines. The meta-analysis included 15 retrospective studies including patients treated for VaIN 2+. Pooled risk ratios (RRs) with 95% confidence intervals (CIs) were calculated for treatment modalities (laser ablation, surgical excision, topical therapy) and clinical risk factors (prior hysterectomy, multifocality, immunosuppression, HPV16 infection and history of cervical intraepithelial neoplasia (CIN)). **Results:** Immunosuppression was significantly associated with a higher risk of treatment failure (RR = 2.01; 95% CI 1.12–3.60; *p* = 0.030). Topical therapies were found to have a significantly higher risk of treatment failure compared to laser ablation (RR = 1.92; 95% CI 1.34–2.92; *p* = 0.009). No statistically significant difference in recurrence risk was found between laser ablation and surgical excision, and between surgical excision and topical therapy. Factors such as prior hysterectomy, multifocality, and history of CIN did not show a statistically significant association with recurrence in the pooled models. **Conclusions:** Immunosuppression is a critical risk factor for VaIN 2+ recurrence, highlighting the need for individualized management and closer surveillance in this population. Surgical and ablative methods appear superior to topical agents in controlling high-grade disease. Given the retrospective nature of current data, standardized prospective studies are required to refine treatment algorithms.

## Simple Summary

High-grade vaginal intraepithelial neoplasia (VaIN 2+) is a rare precancerous condition that can progress to vaginal cancer. Due to its low incidence rate, there is a lack of clear evidence regarding the most effective treatment methods and clinical factors associated with disease recurrence after therapy. This study aimed to combine results from existing research to determine which therapies result in the best outcomes and which patients are at the highest risk of treatment failure. Our analysis of 15 studies involving over 2000 cases found that patients with immunosuppression are twice as likely to experience a recurrence. Additionally, laser-based treatments were found to be more effective than topical therapies. These findings can help clinicians choose the most appropriate treatment strategies and identify high-risk patients who require more frequent follow-up care.

## 1. Introduction

Vaginal intraepithelial neoplasia (VaIN) is a rare disease strongly associated with human papillomavirus (HPV) infection. It occurs in approximately 2–3 per 10,000 women and accounts for approximately 0.4% of precancerous lesions of the lower female genital tract [1,2]. In addition, it is a precursor to vaginal cancer [3]. VaIN is usually asymptomatic and often diagnosed incidentally based on abnormal cytology or histopathology results [4]. There are no screening tests for vaginal cancer and precancerous lesions. VaIN lesions are often difficult to detect and are usually diagnosed after multiple examinations [5]. However, given the increased availability of colposcopy, these lesions are being diagnosed more frequently than before [6]. Colposcopic evaluation of the entire vaginal wall surface is now recommended for all patients diagnosed with abnormal cytology results. Its incidence is thought to be 100 times lower compared to cervical intraepithelial neoplasia (CIN) [7]. Importantly, however, estimations suggest that, in 75% of women, VaIN coexists with either cervical or vulvar cancer [8]. Numerous risk factors for VaIN include HPV infection, smoking, vaginal dysbiosis, previous hysterectomy, postmenopausal period, or immunosuppression [9,10,11,12,13]. The 2020 World Health Organization (WHO) classification marks out two types of squamous intraepithelial lesions (SILs)—low-grade malignancy, which includes VaIN 1, and high-grade malignancy, including VaIN 2 and VaIN 3 [14]. The management of VaIN includes various therapeutic options, including laser ablation, surgical excision, topical treatment, or brachytherapy [15,16]. The progression rate of VaIN 2 is considered to be around 1.4% and 15.4% for VaIN 3 [17]. Moreover, invasive cancer following treatment of VaIN 2+ has been estimated at 3.2% to 5.8%, with a mean time from treatment to progression of 54.6 to 61 months [18].

Numerous studies have investigated the recurrence risk of VaIN based on factors such as treatment modality, lesion focality, HPV infection, and immunosuppression [18,19]. However, the current literature is predominantly limited to small, single-center retrospective cohorts, making it difficult to draw definitive clinical conclusions. Furthermore, quality synthesized evidence regarding treatment failure, specifically in high-grade VaIN, is distinctly lacking. This study aims to address this gap by systematically evaluating risk factors associated with VaIN 2+ treatment failure, consolidating fragmented retrospective data to better inform clinical management.

## 2. Materials and Methods

### 2.1. Search Strategy

A systematic literature search was performed in PubMed, Scopus, Web of Science, and Cochrane Central Register of Controlled Trials databases for articles published with no date restriction. The review was conducted following the Preferred Reporting Items for Systematic Reviews and Meta-Analyses (PRISMA) guidelines (Supplementary Material) mat and the study protocol was logged in the International Prospective Register of Systematic Reviews (PROSPERO) registry (CRD420261306815). The search strategy consisted of combinations of free text and Medical Subject Headings (MeSH) terms, including “vaginal intraepithelial neoplasia”, “VaIN”, “high-grade VaIN”, “recurrence”, “persistence”, “treatment failure”, “laser ablation”, “vaginectomy”, and “risk factors”.

Following the primary search, reference lists of selected studies were manually screened for other eligible publications. The inclusion criteria were randomized controlled trials (RCTs) and observational studies (retrospective and prospective cohorts) written in English that reported recurrence or persistence rates in patients treated for high-grade VaIN (VaIN 2 or 3). Studies were required to provide extractable data on potential predictors of recurrence (e.g., comparisons between treatment modalities, hysterectomy status, or HPV genotype). The exclusion criteria were: (1) studies focusing exclusively on low-grade VaIN; (2) studies regarding invasive vaginal cancer; (3) case reports, case series, and animal studies; and (4) studies lacking sufficient data to construct contingency tables for analysis.

For non-randomized studies of interventions (NRSIs), including retrospective and prospective cohorts, the ROBINS-I tool (Risk Of Bias In Non-randomized Studies—of Interventions) was employed [20]. ROBINS-I assesses seven domains of bias: confounding, selection of participants into the study, classification of interventions, deviations from intended interventions, missing data, measurement of outcomes, and selection of the reported result. The overall risk of bias for each study was classified as “low”, “moderate”, “serious”, or “critical”. Any disagreements regarding study eligibility or risk of bias grading were resolved by consultation with a senior author (B.S.).

### 2.2. Data Extraction

Titles and abstracts were screened independently by two researchers (F.Ł., M.P.). Full-text articles of potentially relevant studies were retrieved and assessed for eligibility. The following information was collected: author’s name, year of publication, country, study design (retrospective/prospective), sample size (total and VaIN 2+ specific), duration of follow-up, and treatment modalities used. In case of missing data, the authors were contacted in order to provide the necessary information.

For the quantitative synthesis, data were extracted on specific risk factors for recurrence, including: type of treatment, prior hysterectomy status, HPV genotype, and lesion focality. Data were recorded as raw numbers of events (recurrence/persistence) and totals in each subgroup to facilitate Risk Ratio (RR) calculations.

### 2.3. Outcomes

The primary outcome was the failure of treatment of high-grade VaIN (defined as histologically confirmed VaIN 2+ detected after an initial period of disease-free status of at least 6 months), disease persistence (failure to achieve regression after primary treatment), or disease progression (defined as an increase in lesion grade or the development of invasive disease).

### 2.4. Statistical Analysis

Meta-analysis was performed using JASP (version 0.95.1, Amsterdam, the Netherlands). For dichotomous outcomes (recurrence vs. cure), pooled RRs with 95% Confidence Intervals (CIs) were calculated using the Mantel–Haenszel method. Due to the anticipated heterogeneity in study designs and populations (e.g., varying follow-up protocols), a random-effects model was applied for all analyses to provide a more conservative estimate.

Heterogeneity among studies was evaluated using the I^2^ statistic, where I^2^ values of 25%, 50%, and 75% represented low, moderate, and high heterogeneity, respectively. A *p*-value of <0.05 was considered statistically significant.

## 3. Results

**Figure 1:**
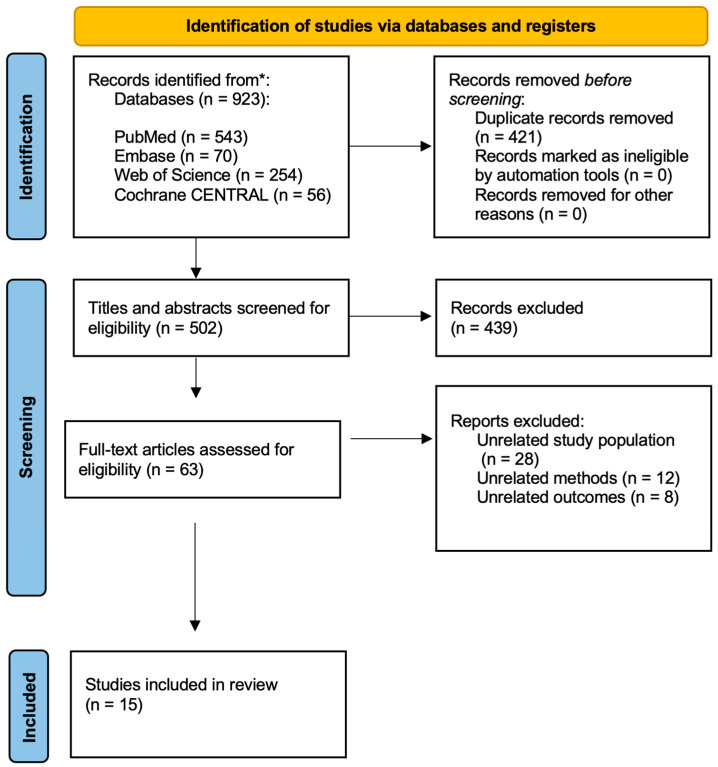
PRISMA flow chart of the screening process.

A total of 923 articles were identified through a systematic review of the literature (Figure 1). After initial screening, 421 duplicates were excluded, and 502 titles and abstracts underwent further screening to evaluate eligibility. A total of 62 publications underwent an in-depth full-text analysis, resulting in 47 studies being excluded from further assessment. Eventually, a total of 15 publications were included in this systematic review (Figure 1).

**Figure 2:**
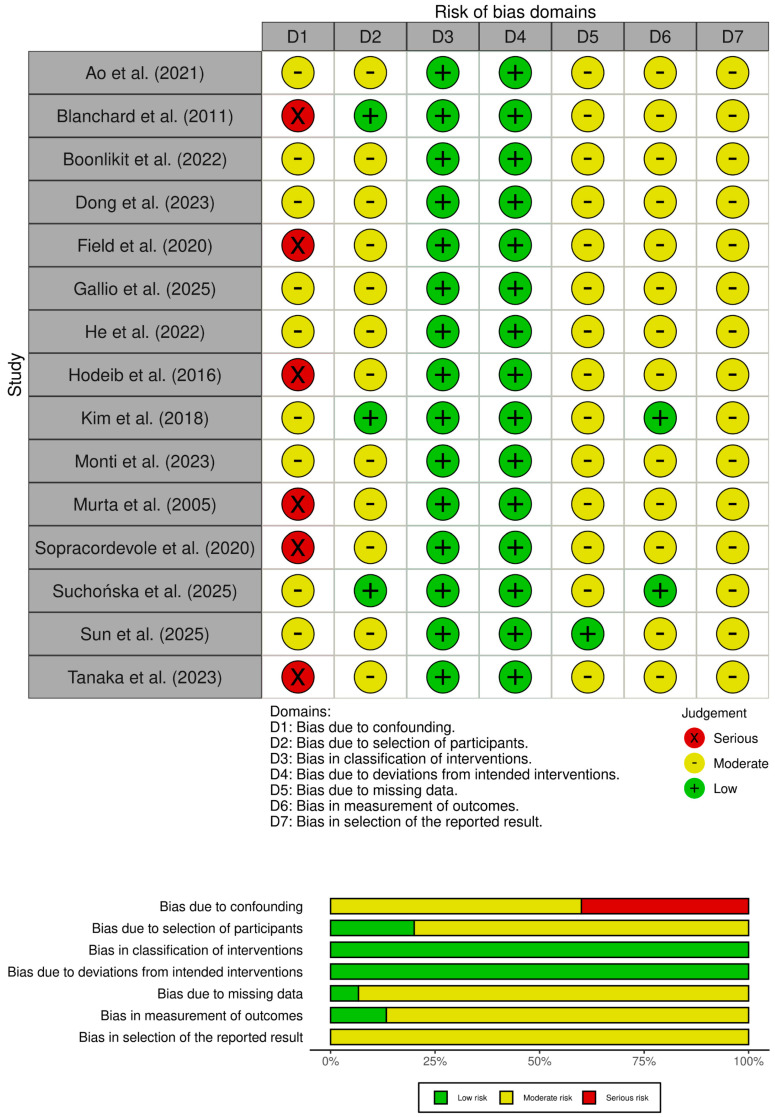
Risk of bias assessment of studies included in the analysis.

The detailed characteristics of the included studies, including study design, population size, and follow-up duration, are summarized in Table 1, and the risk of bias assessment is shown in Figure 2.

### 3.1. Treatment Modality

#### 3.1.1. Laser Ablation vs. Surgical Excision

**Figure 3:**
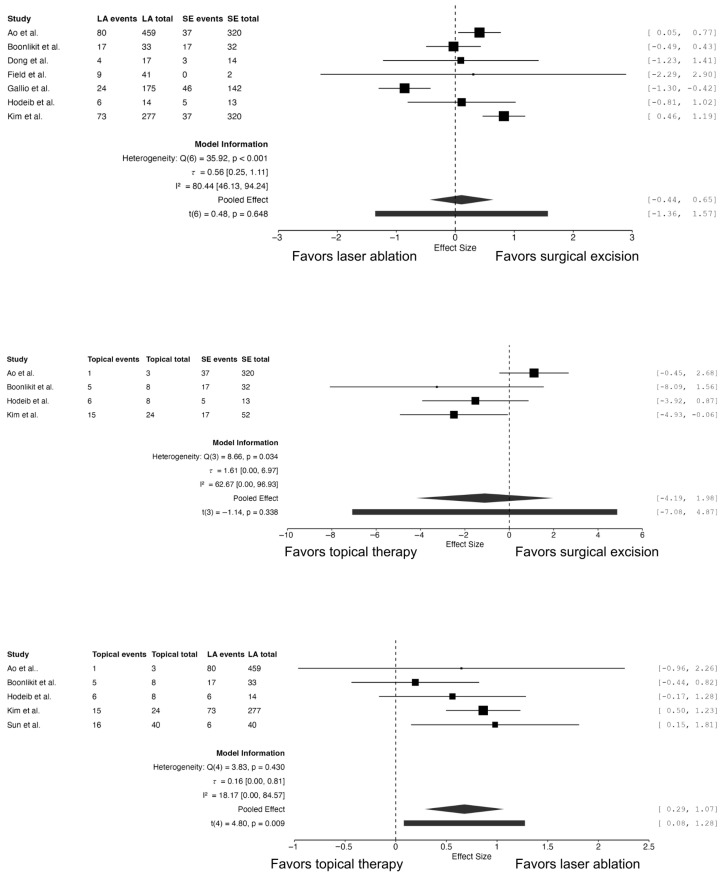
Forest plots comparing treatment failure by therapeutic modality for high-grade VaIN.

Seven studies compared the outcomes of laser ablation and surgical excision in patients with high-grade VaIN [21,23,24,25,26,28,29]. The pooled analysis did not demonstrate a statistically significant difference in recurrence risk between the two treatment modalities (RR = 1.11, 95% CI 0.64–1.92; *p* = 0.648). Substantial heterogeneity was observed across the studies (I^2^ = 80.4%), suggesting variability in patient populations, lesion characteristics, and follow-up protocols (Figure 3).

#### 3.1.2. Topical Therapy vs. Laser Ablation

Four studies evaluated the effectiveness of topical therapies compared with laser ablation [21,23,28,29]. The pooled analysis demonstrated a significantly higher risk of treatment failure among patients treated with topical agents (RR = 1.92, 95% CI 1.34–2.92; *p* = 0.009). Heterogeneity between studies was low (I^2^ = 18.2%), indicating consistent findings across the included cohorts.

#### 3.1.3. Topical Therapy vs. Surgical Excision

Four studies compared topical therapies with surgical excision [21,23,28,29]. The pooled analysis showed no statistically significant difference between the two approaches (RR = 0.90, 95% CI 0.02–7.24; *p* = 0.338). Moderate heterogeneity was observed in this comparison (I^2^ = 62.7%).

### 3.2. Clinical Risk Factors for Treatment Failure

#### 3.2.1. Prior Hysterectomy

**Figure 4:**
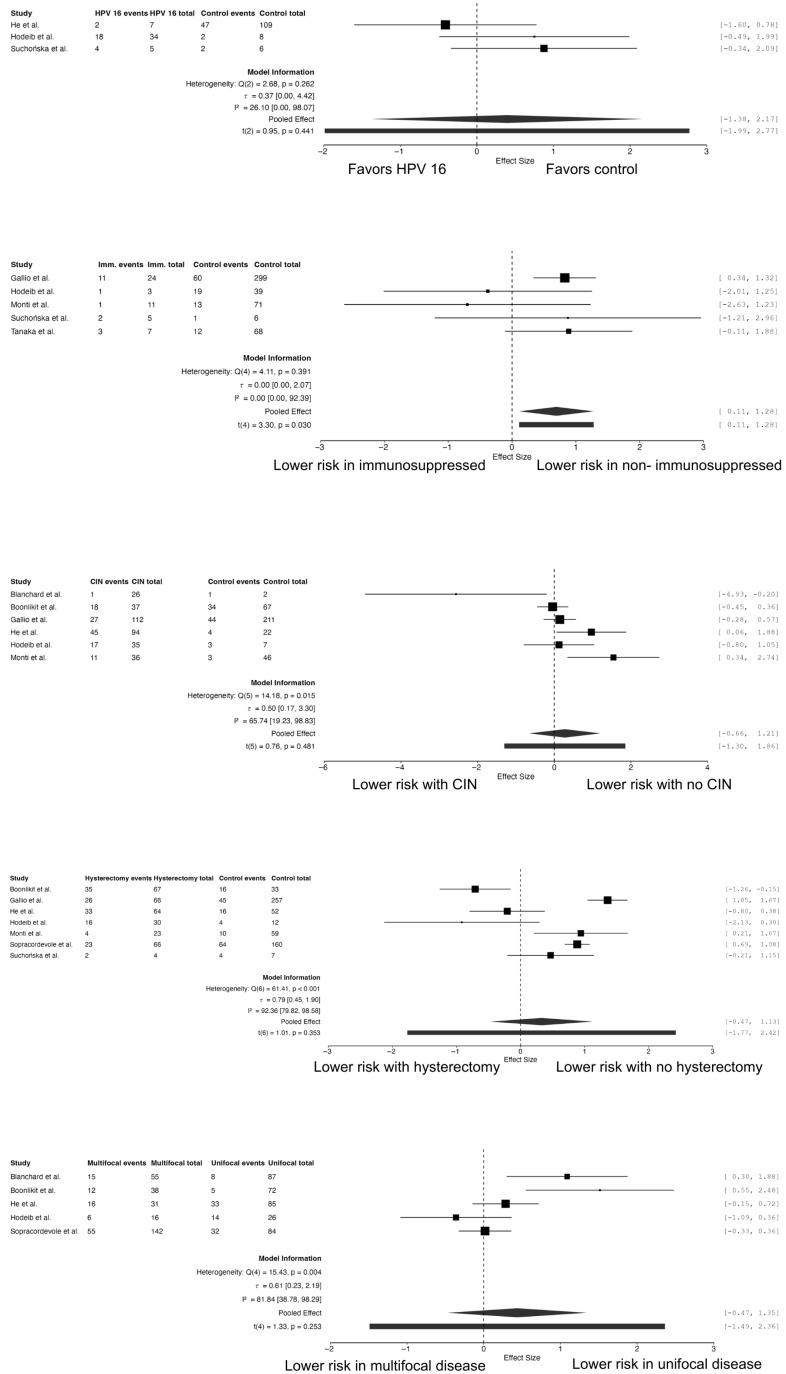
Forest plots of clinical risk factors associated with high-grade VaIN treatment failure.

Seven studies assessed the association between prior hysterectomy and recurrence of VaIN [17,23,26,27,28,30,32]. The pooled analysis did not demonstrate a statistically significant association (RR = 1.38, 95% CI 0.63–3.10; *p* = 0.353). However, substantial heterogeneity was observed across studies (I^2^ = 92.4%), indicating considerable variability in study populations and clinical contexts (Figure 4).

#### 3.2.2. Multifocality

Five studies evaluated the effect of lesion focality on treatment failure risk [17,22,23,27,28]. Although multifocal disease demonstrated a trend toward higher recurrence rates compared with unifocal lesions, the association did not reach statistical significance (RR = 1.55, 95% CI 0.63–3.86; *p* = 0.253). High heterogeneity was observed (I^2^ = 81.8%).

#### 3.2.3. Immunosuppression

Five studies investigated the impact of immunosuppression on treatment outcomes [26,28,30,32,34]. Immunosuppressed patients demonstrated a significantly increased risk of recurrence compared with immunocompetent individuals (RR = 2.01, 95% CI 1.12–3.60; *p* = 0.030). No heterogeneity was detected across studies (I^2^ = 0%).

#### 3.2.4. HPV16 Infection

Three studies analyzed the role of HPV16 infection in the failure of treatment of high-grade VaIN [27,28,32]. The pooled analysis did not demonstrate a statistically significant association between HPV16 positivity and recurrence risk (RR = 1.48, 95% CI 0.25–8.76; *p* = 0.44). Low heterogeneity was observed (I^2^ = 26%).

#### 3.2.5. History of Cervical Intraepithelial Neoplasia (CIN)

Six studies evaluated the impact of a history of CIN on recurrence risk [22,23,26,27,28,30]. The pooled analysis showed no statistically significant association between CIN history and recurrence of VaIN (RR = 1.32, 95% CI 0.52–3.35; *p* = 0.48). Moderate heterogeneity was observed (I^2^ = 65.7%)

## 4. Discussion

To our knowledge, this is the first systematic review and meta-analysis to evaluate treatment outcomes and potential predictors of treatment failure in patients with high-grade vaginal intraepithelial neoplasia (VaIN 2/3).

Due to the rarity of the disease and the limited number of prospective trials, current management strategies remain largely based on retrospective evidence and expert consensus. Our analysis aimed to synthesize the available data to clarify the relative effectiveness of treatment modalities and identify clinical factors associated with treatment failure.

One of the most important findings of our analysis was the significant association between immunosuppression and an increased risk of treatment failure. Immunosuppressed patients had approximately a twofold higher risk of treatment failure than immunocompetent individuals. This observation is biologically plausible, as effective immune surveillance plays a crucial role in clearing high-risk human papillomavirus (hrHPV) infection and controlling HPV-driven intraepithelial lesions [35,36]. Patients with impaired immune function, including those with HIV infection, chronic immunosuppressive therapy, or organ transplantation, may therefore represent a particularly vulnerable population requiring closer surveillance and potentially more aggressive therapeutic approaches [35,36,37].

Another significant finding of our analysis was the higher risk of treatment failure associated with topical therapies compared with laser ablation. While topical agents such as 5-fluorouracil (5-FU) or imiquimod offer the advantage of being non-invasive and preserving vaginal anatomy, their effectiveness may be limited by challenges in achieving consistent drug exposure across the vaginal epithelium and by issues related to treatment adherence [18]. Local irritation, prolonged treatment duration, and variable penetration of topical agents may contribute to the reduced efficacy observed in our pooled analysis [18,32]. These findings suggest that, especially in patients with extensive multifocal disease or those unfit for surgery, they should not be used as primary treatment for high-grade lesions.

In contrast, several factors traditionally considered strong predictors of recurrence did not reach statistical significance in our pooled analysis. Prior hysterectomy, multifocal disease, HPV16 infection, and a history of CIN all demonstrated trends toward increased recurrence risk but were associated with wide confidence intervals and substantial heterogeneity across studies. These findings likely reflect the limited sample sizes and methodological variability among the included studies. However, the considerable heterogeneity observed in this comparison suggests that additional factors, such as surgical technique, lesion location, and follow-up protocols, may substantially influence the outcomes.

Furthermore, the comparison between laser ablation and surgical excision did not reveal a statistically significant difference in the risk of treatment failure. Although excisional techniques provide the advantage of histopathological assessment and margin evaluation, ablative methods preserve vaginal anatomy and may be associated with fewer surgical complications [18,38]. The absence of a clear superiority of either modality in our analysis supports the current clinical practice of individualizing treatment selection based on lesion characteristics, patient comorbidities, and surgeon expertise [18,38]. However, the high heterogeneity observed across studies suggests that further well-designed comparative studies are needed to clarify the relative effectiveness of these approaches.

When considering these individualized strategies, the role of conservative management must also be addressed. While expectant management is widely accepted as the standard of care for low-grade VaIN due to its high rate of spontaneous regression, high-grade VaIN generally mandates active therapeutic intervention due to its significant malignant potential [18]. Conservative management of high-grade lesions is rarely recommended and is typically reserved for exceptional clinical scenarios, such as during pregnancy or in patients with severe surgical contraindications, provided that strict, highly compliant colposcopic and cytological surveillance can be guaranteed [18].

The relationship between HPV16 infection and treatment failure also warrants further investigation. Although HPV16 is the most oncogenic HPV genotype and is strongly associated with cervical and vaginal carcinogenesis, our analysis did not demonstrate a statistically significant increase in recurrence risk among HPV16-positive patients [18]. This may be due to the limited number of studies reporting genotype-specific outcomes and the lack of standardized HPV testing protocols across cohorts. Future research incorporating comprehensive HPV genotyping and viral persistence analysis may help clarify the prognostic significance of specific HPV subtypes in VaIN.

Overall, the findings of this study highlight the complexity of managing high-grade VaIN and underscore the need for individualized treatment strategies. Given the rarity of the disease, collaborative multicenter studies and prospective registries will be essential to improve the quality of evidence and refine treatment algorithms.

The main strength of this study lies in the comprehensive synthesis of available evidence regarding treatment outcomes and risk factors for failure of treatment in high-grade VaIN. By focusing specifically on VaIN 2/3 lesions and pooling data from multiple cohorts, we were able to provide a broader overview of clinical trends that individual studies may not have been adequately powered to detect. Moreover, our systematic review and meta-analysis were conducted in accordance with the strict methodological requirements of the PRISMA checklist.

Nevertheless, several limitations should be acknowledged. First, all of the included studies were retrospective in design, which introduces the potential for selection bias and confounding. Second, definitions of recurrence and persistence varied slightly between studies, particularly with regard to follow-up duration. Third, considerable heterogeneity was observed in several analyses, likely reflecting differences in patient populations, treatment protocols, and surveillance strategies. Furthermore, while subgroup or sensitivity analyses (e.g., stratifying by specific follow-up durations, lesion sizes, or surgical margins) would be highly valuable to explore this heterogeneity, the limited number of studies available for each comparison and the lack of standardized reporting across the primary literature precluded robust subgroup pooling. Finally, data regarding HPV vaccination status and viral persistence were largely unavailable, limiting our ability to evaluate the potential impact of vaccination on recurrence risk.

## 5. Conclusions

This systematic review and meta-analysis provide an updated synthesis of treatment outcomes and risk factors for treatment failure in patients with high-grade vaginal intraepithelial neoplasia.

**Figure 5:**
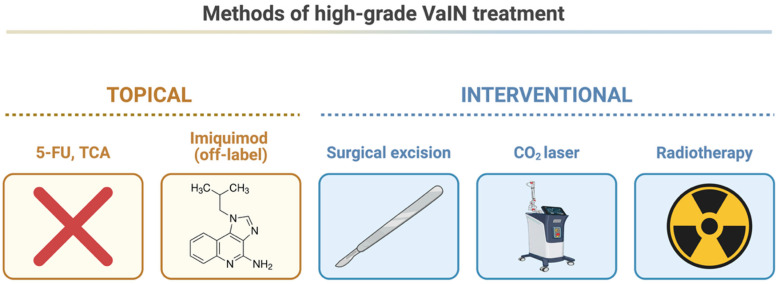
Summary of high-grade VaIN treatment modalities. Created with biorender.com.

Our analysis demonstrated that immunosuppression is a significant risk factor for treatment failure, highlighting the importance of intensified surveillance and individualized management in this high-risk population. In addition, topical therapies were associated with higher rates of treatment failure compared with laser ablation, suggesting that surgical or ablative approaches may provide more reliable disease control for high-grade lesions (Figure 5).

Future studies should focus on standardized outcome definitions, incorporation of HPV genotyping and viral persistence monitoring, and the evaluation of emerging therapeutic strategies, including the potential role of HPV vaccination in reducing recurrence risk.

Based on the results of our meta-analysis and the ESGO, ISSVD, ECSVD, and EFC consensus statement [18], surgical or ablative methods may provide more reliable disease control for high-grade lesions compared to topical therapies. However, given the retrospective nature of current data, standardized prospective studies are required to refine and solidify treatment algorithms. We recommend either laser ablation or surgical excision as the primary treatment, given the lowest risk of treatment failure.

5-FU—5-fluorouracil; CO_2_—carbon dioxide; TCA: trichloroacetic acid; VaIN—vaginal intraepithelial neoplasia.

## Figures and Tables

**Table 1:** Characteristics of studies included in the analysis.

Study (Author, Year)	Country	Study Design	Population (N)	High-Grade VaIN (N) *	Treatment Modalities Analyzed	Follow-Up (Months) **
Ao et al. (2021) [21]	China	Retrospective	1478	832	Laser ablation, surgical excision, ALA-PDT	Mean: 34.5; Median: 14
Blanchard et al. (2011) [22]	France	Retrospective	28	28	Laser ablation	Median: 41
Boonlikit et al. (2022) [23]	Thailand	Retrospective	104	104	Laser ablation, surgical excision, topical	Median: 23.6
Dong et al. (2023) [24]	China	Retrospective	175	40	Laser ablation, surgical excision, PDT	Median: 8
Field et al. (2020) [25]	UK	Retrospective	88	53	Laser ablation, surgical excision	Median: 26
Gallio et al. (2025) [26]	Italy	Retrospective	323	323	Laser ablation, surgical excision	Mean: 62.43
He et al. (2022) [27]	China	Retrospective	116	116	Laser ablation	Mean: 49.5
Hodeib et al. (2016) [28]	USA	Retrospective	42	42	Laser ablation, surgical excision, topical	Median: 64
Kim et al. (2018) [29]	South Korea	Retrospective	576	397	Laser ablation, surgical Excision, topical	Median: 44.6
Monti et al. (2023) [30]	Italy	Retrospective	82	82	Laser ablation, surgical excision	Median: 15.5
Murta et al. (2005) [31]	Brazil	Retrospective	38	38	Laser ablation, surgical excision, topical	Range: 6–60
Sopracordevole et al. (2020) [17]	Italy	Retrospective	226	226	Laser ablation, surgical excision	Median: 42.8
Suchońska et al. (2025) [32]	Poland	Retrospective	24	11	Laser ablation, surgical excision, topical	Median: 12.3
Sun et al. (2025) [33]	China	Retrospective	125	125	Laser, ALA-PDT	Median: 5
Tanaka et al. (2023) [34]	Japan	Retrospective	647	647	Laser ablation	Median: 48

ALA-PDT—5-aminolevulinic acid photodynamic therapy; * Note: Where total study population included Low-Grade VaIN, the N listed reflects the High-Grade (VaIN 2/3) subgroup used for extraction. ** Follow-up is expressed as Median or Mean (months), depending on study reporting.

## Data Availability

Datasets generated in this study are available from the corresponding author upon reasonable request.

## Supplementary Materials

The following supporting information can be downloaded at: https://www.mdpi.com/article/10.3390/cancers18081302/s1, PRISMA 2020 Checklist. Reference [39] is cited in the Supplementary Materials.

## Abbreviations

The following abbreviations are used in this manuscript:

5-FU	5-fluorouracil
ALA-PDT/PDT	5-aminolevulinic acid photodynamic therapy/Photodynamic therapy
CI	Confidence interval
CIN	Cervical intraepithelial neoplasia
CO_2_	Carbon dioxide
ECSVD	European College for the Study of Vulval Disease
EFC	European Federation for Colposcopy
ESGO	European Society of Gynaecological Oncology
HIV	Human immunodeficiency virus
HPV	Human papillomavirus
hrHPV	High-risk human papillomavirus
ISSVD	International Society for the Study of Vulvovaginal Disease
LA	Laser ablation
MeSH	Medical Subject Headings
NRSI	Non-randomized studies of interventions
PRISMA	Preferred Reporting Items for Systematic Reviews and Meta-Analyses
PROSPERO	International Prospective Register of Systematic Reviews
RCT	Randomized controlled trial
ROBINS-I	Risk Of Bias In Non-randomized Studies—of Interventions
RR	Risk ratio
SE	Surgical excision
SIL	Squamous intraepithelial lesion
TCA	Trichloroacetic acid
VaIN	Vaginal intraepithelial neoplasia
WHO	World Health Organization
